# BioExcel Building Blocks, a software library for interoperable biomolecular simulation workflows

**DOI:** 10.1038/s41597-019-0177-4

**Published:** 2019-09-10

**Authors:** Pau Andrio, Adam Hospital, Javier Conejero, Luis Jordá, Marc Del Pino, Laia Codo, Stian Soiland-Reyes, Carole Goble, Daniele Lezzi, Rosa M. Badia, Modesto Orozco, Josep Ll. Gelpi

**Affiliations:** 1 Barcelona Supercomputing Center (BSC), Jordi Girona 29, 08034 Barcelona, Spain; 2 Institute for Research in Biomedicine (IRB Barcelona), The Barcelona Institute of Science and Technology (BIST), Baldiri Reixac 10, Barcelona, 08028 Spain; 3 School of Computer Science, The University of Manchester, Manchester, United Kingdom; 4 Department Biochemistry and Molecular Biomedicine, University of Barcelona, Barcelona, Spain

**Keywords:** Computational platforms and environments, Data processing

## Abstract

In the recent years, the improvement of software and hardware performance has made biomolecular simulations a mature tool for the study of biological processes. Simulation length and the size and complexity of the analyzed systems make simulations both complementary and compatible with other bioinformatics disciplines. However, the characteristics of the software packages used for simulation have prevented the adoption of the technologies accepted in other bioinformatics fields like automated deployment systems, workflow orchestration, or the use of software containers. We present here a comprehensive exercise to bring biomolecular simulations to the “bioinformatics way of working”. The exercise has led to the development of the BioExcel Building Blocks (BioBB) library. BioBB’s are built as Python wrappers to provide an interoperable architecture. BioBB’s have been integrated in a chain of usual software management tools to generate data ontologies, documentation, installation packages, software containers and ways of integration with workflow managers, that make them usable in most computational environments.

## Introduction

Biomolecular simulations have attained in the last years a level of maturity that allows to use them as “computational microscopes” to gain insight in biological processes. Atomistic simulations extend now to the μs range, approaching the time range of biological processes^1,2^. Coarse-grained simulations can go even further, in the length of simulations, and the size of the systems that can be analysed^3–6^. The traditional scope of simulations has overpassed the single protein or small nucleic acid systems to deal with relevant multiprotein and protein-nucleic acid complexes, nucleosomes, long segments of RNA, sections of chromatin or even full chromosomes^5^. This scenario envisions now a clear bridge between biomolecular simulations and genomics. Multiscale approaches can now bring together, for instance, Chip-seq data with simulation of protein-DNA complexes, or HiC or oligopaint FISH experiments with large scale simulations of chromatin fibers^5^. However, the type of tools, and the way they are used differ between genomics and biomolecular simulations. Simulations have been traditionally based on a reduced number of well optimized codes run in HPC systems, where they indeed occupy a large amount of resources (over 60 M CPU-hours of BSC’s MareNostrum supercomputer were dedicated to biomolecular simulations in 2018). On the other hand, traditional bioinformatics uses many competing tools usually orchestrated in complex workflows. Considering data, genomics mobilizes indeed the major amount of it, however, the storage of a typical μs-range trajectory on a mid-sized system requires already some hundreds of GB like a human whole genome obtained by Next-Generation Sequencing (NGS).

Workflow orchestration is a well-accepted concept in bioinformatics. No single, universal, solution exists, and the number of available frameworks to build and run workflows is large (https://github.com/common-workflow-language/common-workflow-language/wiki/Existing-Workflow-systems). Initiatives in the past like myGrid^7^ and BioMoby^8^, or more recent initiatives like CWL^9^, or WDL (https://software.broadinstitute.org/wdl/), have attempted to define an interoperable ecosystem to run bioinformatics tools, web-services and the workflows made out of them. Managers like Taverna^10^, Rabix^11^, Cromwell (https://cromwell.readthedocs.io/en/stable/), KNIME^12^ or repositories like myExperiment^13^ allow to execute or store workflow definitions for further re-usage. In this context, the ELIXIR (http://elixir-europe.org) organization is working to put in place recommendations to organize such ecosystem. At the level of registration, bio.tools^14^ and Fairsharing^15^ provide repositories for tools and standards. Specification languages like openAPI (https://www.openapis.org/), and CWL^9^ are being recommended to document APIs and workflows, respectively. In terms of workflow execution, Galaxy^16^ appears as the most popular framework, although other managers are also commonly used (e.g. Nextflow^17^, PyCOMPSs^18^, Snakemake)^19^. To formalize this scenario, the FAIR principles^20^, initially presented to improve the quality of scientific data, are now being extended to research software. The key requirements for that (registries, standards, software managers and open repositories) are already available. Several organizations including the Software Sustainability Institute (https://www.software.ac.uk/), Research Software Engineers’ associations, or ELIXIR itself are participating actively in the discussion.

Bioinformatics initiatives have little application to the simulation world. Simulations themselves are run in HPC systems in highly optimized environments. Most of the work, like setting up the simulation, a key step to assure the quality of the results^21–24^, and the analysis of trajectories, is done almost manually. Modelers use *in-house* scripts, typically based on the software included in the simulation packages. In this situation, researchers usually limit themselves to a single package for all steps: setup, simulation, and analysis. Therefore, since the possibility of complementing software functionalities across packages is limited, developers should provide complete sets, re-implementing what other packages provide already. Additionally, since data formats are also diverse, data conversion modules proliferate, what in turn raises the question of which combinations of tools (although theoretically compatible) would give correct scientific answers.

Efforts to automate simulation setup and analysis do exist. Several graphical interfaces have been designed to ease the interaction with specific simulation packages^25–30^. These tools are especially useful for non-experts as they simplify the learning process. However, these utilities are still linked to specific simulation packages. One of the attempts, by our group, was MDWeb^31^. This was the first approach to offer a unified workbench allowing to setup a protein system for atomistic molecular dynamics simulation, able to work for GROMACS^32^, NAMD^33^, and Amber^34^, three of the most popular simulation packages. Remarkably, MDWeb is powered internally by a series of web services built within the BioMoby framework and uses a common ontology of data types for the three simulation packages (http://mmb.irbbarcelona.org/MDWeb2/help.php?id=ontology). In this sense, this attempt, still in use with over 3,000 registered users, was rather unique. MDWeb was extended to the nucleic acids world with a nucleic-acids specific analysis portal, NAFlex^35^. At the large-scale end, systems have been designed to manage large scale simulation projects. Copernicus^36^ combines peer-to-peer communication strategies with a simulation specific workflow management system, able to control large simulation sets in a distributed computational network. The iBIOMES project^37,38^ reported an infrastructure to manage and share distributed simulation data, based in the iRODS framework (https://irods.org/). iBioMES has been used recently to manage nucleosome simulation data^39^, in a clear example for the growing overlap between simulation and genomics. Some simulation databases have also been built. Dynameomics^40^, centered in analysing protein folding and stability, MoDEL^22^ offering an initial attempt of covering a significant sample of known protein structures, and BigNASim^23^, specialized in Nucleic Acids. Remarkably, MoDEL and BigNASim provided ontologies for representing simulation data (https://mmb.irbbarcelona.org/BIGNASim/help.php?id=onto).

Even though a large set of tools are normally combined, the concept of workflow, as understood in general bioinformatics, is of limited usage. As said, most systems are setup and analyzed using *in-house* scripts. Recently, the BioExcel Center of Excellence (http://bioexcel.eu) has taken the objective of pushing the concept and usage of workflows into the biomolecular research field. In this work, we present a comprehensive exercise joining ELIXIR’s recommendations and services, FAIR principles, and biomolecular simulations. We have selected the automatic setup for molecular dynamics simulations of a protein system including sequence variants, as case for demonstration. The aim of the exercise is to assess the feasibility of working according the FAIR principles and ELIXIR’s recommendations in a field that is considered out of the scope of common bioinformatics. We will present a fully interoperable software library (the BioExcel Building Blocks, BioBBs) based mainly on (but not limited to) GROMACS^32^ software components. For the deployment of BioBBs, we have leveraged existing platforms and services commonly used in bioinformatics, like BioConda^41^, BioContainers^42^ or Galaxy^16^. Workflows built using components of such library have been executed in several complementary computational environments, including personal desktops, virtualized systems, public e-infrastructures, and HPC systems. Besides, the components are documented using CWL and openAPI, what opens the possibility of run them in CWL complaint workflow managers.

## Results and Discussion

### Moving toward  FAIR principles

FAIR principles^20^ were defined with the aim of improving the quality of bioinformatics data repositories. Main principles include (1) *Findability:* Data should be findable, i.e. identified by permanent identifiers and included in searchable registries; (2) *Accessibility*: Data should be stored in permanent repositories and accessible in a machine readable form, (3) *Interoperability*: Data should use well-documented formats and standards to allow to interoperate with complementary datasets; and (4) *Reusability*: Documentation about the conditions and limitations of data reusability should be provided. Adherence to these principles has become part of the best-practices in bioinformatics data management and begins to be generally understood and accepted by the research community. They cannot be applied blindly to research software, but the general guidelines can be adapted.

#### Findability

A primary requirement for findability in the case of software is the availability of a software registry. Traditional software repositories like GitHub (https://github.com), are suitable for such usage although they are not usually seen as data resources, and the amount of available scientific metadata is limited. To overcome this limitation, registries with different degrees of acceptance exist (https://www.genscript.com/tools.html; https://omictools.com/; https://www.fda.gov/ScienceResearch/BioinformaticsTools/default.htm). ELIXIR has pushed its own tools registry (bio.tools)^14^. It includes a large set of metadata that allows to search for tools according to their scientific utility, and provides extended metadata regarding publications, documentation and support. It is linked to ELIXIR’s software benchmarking platform, openEBench (https://openebench.bsc.es), which in turn provides data for technical and scientific quality assessment of bioinformatics applications. One of the most remarkable features of bio.tools is the use of an extended ontology (EDAM^43^) for annotation. EDAM annotations allow to classify tools according the type of data they consume or produce and provides a controlled vocabulary to define their precise functionality. This information has been used to derive tools’ annotation for CWL, or Galaxy^44,45^ automatically. Unfortunately, ontology terms for structural bioinformatics, in general, and biomolecular simulation specifically, were scarce in EDAM. The generation of ontologies on simulation have been attempted in the past^22,23^, but such ontologies have been seldom used outside the projects that generated them. However, the interest for addressing simulation data management has increased recently^24^.

The first step of this exercise was to essay the registration in bio.tools of tools required for setup and analysis of a protein simulation. From this assay, several missing data types, file formats, and functionalities were collected (see Supplementary Table S1). We have taken the experience in MDWeb^31^, MoDEL^22^, and BigNASim^23^ ontologies to fill the gaps in EDAM. The additions included setup, simulation and analysis operations, specific data types like system topology, trajectories, or principal components, and file formats covering the most popular simulation codes (Supplementary Table S1). These new terms have been already included in EDAM v1.22 (https://raw.githubusercontent.com/edamontology/edamontology/master/EDAM_dev.owl) and will be available for tools annotation in short. More than thirty simulation related tools, besides the BioBB library components have been registered in bio.tools. To provide an additional means for findability, a BioSchemas – based specification (http://bioschemas.org/) has been included in the appropriate places of BioBB’s documentation.

#### A software architecture for interoperability

The recipe for full tool interoperability is theoretically simple: the use of a common, universal, data model. Past attempts like myGrid^7^ and BioMoby^8^ put foundations to this concept, by building a community-based data ontology and suggesting tool developers to stick to it when generating new tools. However, this attempt was not successful. The community-based approach made difficult to keep control on the evolution of the ontology. Similarly, in Galaxy servers, for instance, system administrators may add *ad-hoc* types and formats, hence contributing to make the scenario even more complex. In summary, attempting to generate a common data model for bioinformatics remains as a hard issue. Fortunately, when we focus on specific fields (NGS, array analysis, etc.), the options are limited, and *de-facto* standards do exist (bam, vcf, gff file formats in NGS analysis, for instance). Similarly, in biomolecular simulation the limited number of software packages makes the scenario less complicated. In any case, however, tool interoperability is an issue; a large set of operations in bioinformatics are, in fact, format conversions, and there is no security that an input data file is compatible with a given tool, even though that the format is the correct one.

**Fig. 1:**
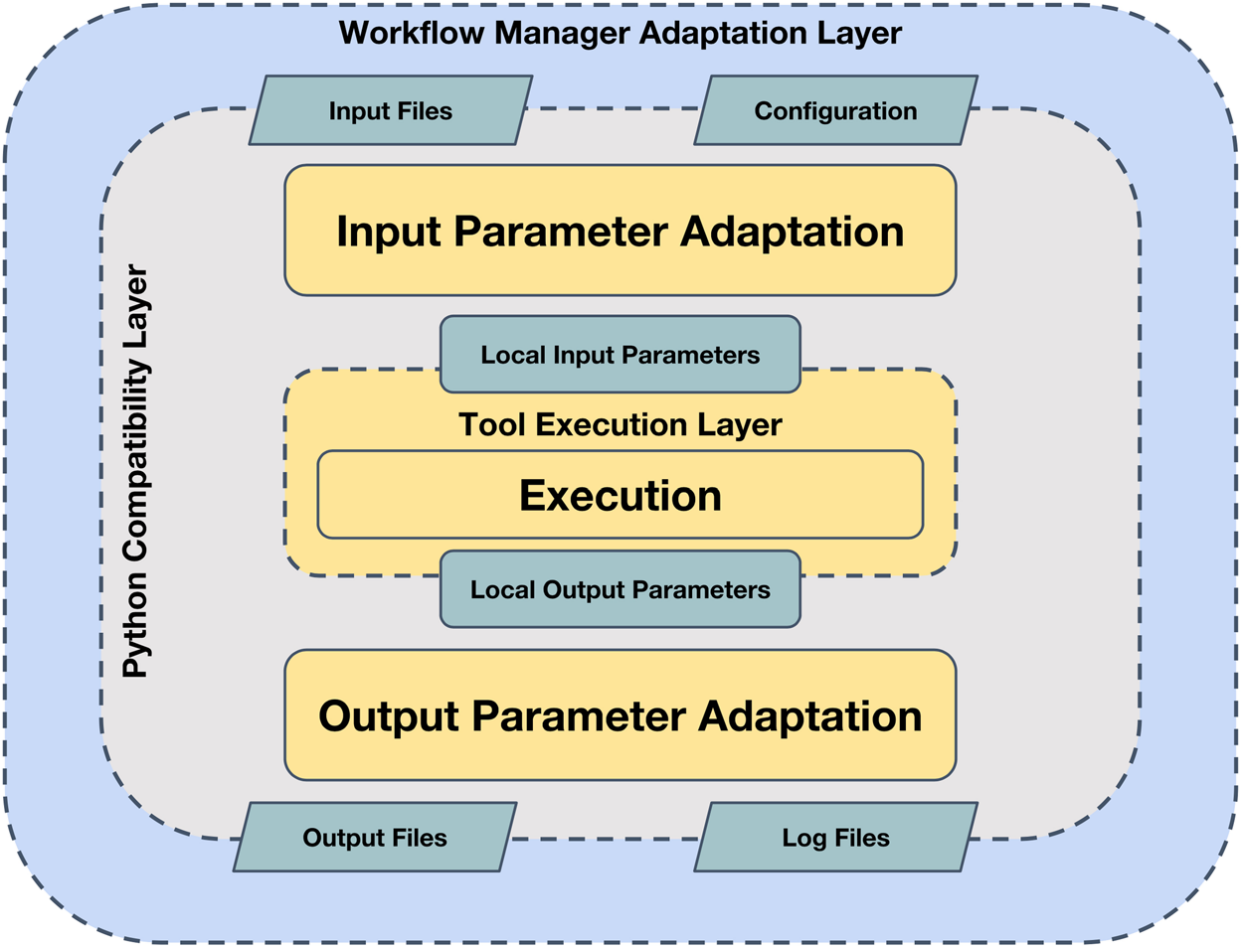
BioExcel building block architecture. BioBB’s structure split in three main layers: The inner layer corresponds to the original tool unaltered, the second one, the Python compatibility layer provides a standardized interface, the third one, the outer workflow manager adaptation layer translates the Python standard interface to each specific WF manager.

In this exercise, we have defined a specific software architecture to contribute to the interoperability (Fig. 1). We use simple wrappers, written in Python, to encapsulate software components. Wrappers are organized in layers. The inner layer corresponds to the original tool, unaltered. Command-line tools, web services, software containers, or even remote calls to HPC systems, can be included here. A second one, *the compatibility layer*, provides the module with a well-defined interface for input, output, configuration, and provenance. It performs internally the necessary format conversions at input and output and launches the tool. This interface can be fully documented and specified using accepted standards like openAPI or CWL and can remain stable even when the associated tool needs to be updated. These two-layer wrappers can be already integrated in scripts as Python modules or executed as standalone command-line tools. A third layer, *the adaptor*, may be required for the integration in execution engines or e-infrastructures. BioBBs adaptors for Galaxy, PyCOMPSs, and CWL compliant managers are provided. Such adaptors can be used as templates to extend the usability of the library to other environments.

This architecture, even though it does not provide a common data model, do provide a uniform and stable interface, with enough information to plug the components into interoperable workflows (see below). Besides, any updates in the inner software tool would require only to update the wrapper, maintaining compatibility with previous versions, workflows, and with the chosen deployment options. Table 1 shows the present list of BioBBs with indication of their functionalities and associated tools.

**Table 1:** List of available BioExcel Building blocks.

Block group	Block Id	Wrapped software	Functionality description
biobb_io	MmbPdb	API Call	Downloads a PDB file from the RCSB or MMB REST APIs
	MmbPdbVariants	API Call	Creates a text file containing a list of all the variants mapped to a RSCB PDB code from the corresponding UNIPROT entries.
	MmbPdbClusterZip	API Call	Creates a zip file containing all the PDB files in the given sequence similarity cluster percentage of the given RSCB PDB code
biobb_model	fix_side_chain	in house	Reconstructs the missing side chains and heavy atoms of the given PDB file
	mutate	in house	Creates a new PDB file performing the mutations given in a list of amino acid mutations to the input PDB file.
biobb_md	Pdb2gmx	gmx pdb2gmx	Creates a compressed (ZIP) Gromacs topology (TOP and ITP files) from a given PDB file.
	Editconf	gmx editconf	Creates a Gromacs structure file (GRO) adding the information of the solvent box to the input structure file.
	Genion	gmx genion	Creates a new compressed Gromacs topology adding ions until reaching the desired concentration to the input compressed Gromacs topology.
	Genrestr	gmx genrestr	Creates a new Gromacs compressed topology applying the indicated force restrains to the given input compressed topology.
	Grompp	gmx grompp	Creates a Gromacs portable binary run input file (TPR) applying the desired properties from the input compressed Gromacs topology.
	Mdrun	gmx mdrun	Performs molecular dynamics simulations from an input Gromacs TPR file.
	Make_ndx	gmx make_ndx	Creates a Gromacs index file (NDX) from an input selection and an input Gromacs structure file.
	Solvate	gmx solvate	Creates a new compressed Gromacs topology file adding solvent molecules to a given input compressed Gromacs topology file.
	Ndx2resttop	in house	Creates a new Gromacs compressed topology applying the force restrains to the input groups in the input index file to the given input compressed topology.
biobb_analysis	cluster	gmx cluster	Creates cluster structures from a given input trajectory.
	rms	gmx rms	Performs an RMS analysis of the given input trajectory.
	cpptraj	cpptraj	Performs multiple analysis of a given trajectory.
biobb_common	—	—	BioBB Base structure & common elements
biobb_template	—	—	Generic template to build new blocks

Blocks are grouped by the type of operation and external tool.

#### Providing accessibility and enabling (re)usability

**Fig. 2:**
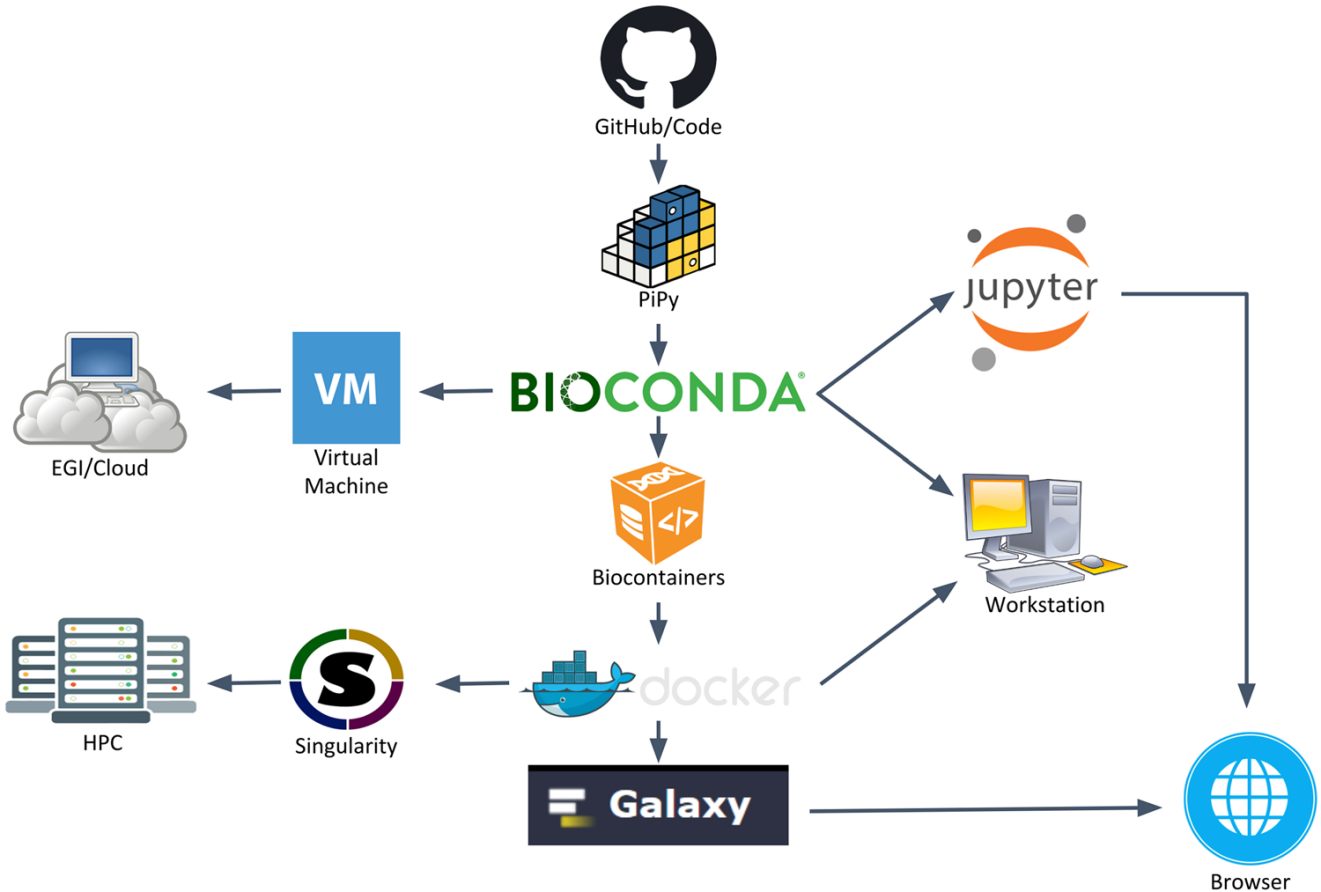
Recommended distribution and deployment flow of the BioBBs. Distribution and packaging tools used to facilitate BioBB’s installation and execution in a wide range of platforms: HPC, Cloud computing, user workstations and even browser interfaces.

In the case of tools, the *accessibility* requirement is even stricter than for data: software not only should be accessible, it needs to be installed and executed. Different execution scenarios should be considered in the case of biomolecular simulations. They include personal workstations, used mainly for setup and analysis, or HPC systems where simulations are usually obtained. To address this principle, BioBB’s use several deployment possibilities. Figure 2 shows a global information flow, and Online-only Table 1 summarizes the URLs corresponding to the different BioBB deployment alternatives. The main software repository used is available on Github. Information embedded in the code allows to generate (1) documentation using the ReadTheDocs platform (https://readthedocs.org/), (2) a JSON schema for library specification using openAPI, and (3) a reference CWL specification. To ease the deployment in a complete set of environments we have put together several packaging systems and services (Fig. 2). From the code deposited in Github, BioBBs have been uploaded to the Python Packaging Index Pypi (https://pypi.org/). Also, BioConda^41^ packages have been prepared. These will allow to handle software dependencies in a transparent way, including the installation of the embedded tools. Considering only these two options, the package would be already available for installation where command-line is the main execution procedure, like personal workstations, clusters, virtual machines, or HPC. Installation can be done both as system-wide Python packages or using Python virtual environments. This kind of installation is illustrated by the execution of the lysozyme test (see below) in a Jupyter Notebook (https://jupyter.org/). Following from BioConda packages, and due to its integration with the BioContainers project^42^, Docker containers are automatically generated and deposited in the quay.io repository. Offered Docker containers provide functionality for either individual packages to be integrated in more complex layouts, or complete workflows. Docker containers, in turn, are converted to Singularity containers that can be used in security demanding environments like HPC. Containers allow the non-expert user to deploy the software easily. For instance, Docker containers have been used to deploy BioBBs in a test Galaxy installation (http://dev.usegalaxy.es). BioBBs, encapsulated as Virtual Machines, are also available on BioExcel cloud portal (https://bioexcel.ebi.ac.uk), and EGI’s appDB (https://appdb.egi.eu). Table 2 summarizes the recommended installation and execution options for the environments tested in the project.

**Table 2:** (A)vailable and (R)ecommended alternatives for Biobb installation and workflow execution.

Architecture	Installation alternatives				Workflow Execution alternatives			
	PyPI	BioConda	VM	Container	Script	CWLtool	PyCOMPSs	Galaxy
Workstation	A	R (T)	A	A	R (T)	A	A	
Cloud	A	A	R (T)	R	R (T)	A	A	
MareNostrum (HPC)	A	A (T)		R	A		R (T)	
Galaxy		A		R (T)				R (T)

(T)est executions performed. Container generic denomination corresponds to Docker containers in the workstation, cloud and Galaxy cases and to Singularity containers in the MareNostrum HPC case.

BioBBs are fully open source, distributed under the Apache-2 license. Wrapped applications have their own licensing schemes, but for the library provided at present only open source software has been included.

#### Testing BioBBs in several environments. Setup for simulation for protein variants workflow

To test the feasibility of the software architecture, we have chosen a well-known procedure, the setup in standard conditions for molecular dynamics simulations of a protein system with sequence variants. We have used two biological systems: Lysozyme (PDB id 1AKI)^46^, and Pyruvate kinase (PDB id 2VGB)^47^. Lysozyme is a small protein (129 res), which structure is available at a high resolution. The second system, Pyruvate kinase is a 200 kDa homo-tetramer, meaning a ~400,000 atom system after setup. Pyruvate kinase is a well-studied system with relevance in the understanding of allosteric regulation, but also of biomedical interest: more than 200 sequence variants related to pathogenic effects have been reported^48^. The test-cases consisted in a standard setup for NPT simulation with explicit solvent of several selected variants, followed with 5 ns long simulations, and a simple RMSd comparative analysis (see Method section). Supplementary Figs S1 and S2 show a schema of the simulation setup workflow as rendered by CWL viewer (https://view.commonwl.org) and Galaxy respectively. We have tested (1) the feasibility of running the workflow (including software installation, and workflow execution) in a variety of computational environments (Lysozyme test) and (2) its scalability on HPC systems (Pyruvate kinase test). Supplementary Table S3 shows a summary of the architectures and the executions performed. Execution times are shown just for illustration purposes and are totally dependent on the hardware used. Since most of the execution time corresponds to the simulation phases, no significant overhead in using the different execution approaches was detected. Parallelization has been carried out at different levels. PyCOMPSs has been used to deal with simulations of different protein variants, at a ratio of 1 variant per process. GROMACS parallelization schemes (OpenMP for intra-node parallelization and MPI when several nodes were involved) were used in the simulation phase. Linear scaling has been observed in all cases (note the similar wall-clock times between the two extreme executions made at BSC’s MareNostrum, ranging from 2 variants, 384 cores, to 200 variants, 38,400 cores).

## Conclusions

Biomolecular simulations are seldom considered as part of the field known as bioinformatics, even structural bioinformatics. Reasons for that come not only from the use of a different kind of tools and computational resources, but also from the traditional lack of applicability of simulation results to day-to-day biology. In the recent years, simulation has attained a significant level of maturity, and simulation results are now compatible with biologically relevant systems and time scales. Biomolecular simulations are already tackling questions that can be relevant for genomics, or transcriptomics. However, the isolation of biomolecular simulations in the context of bioinformatics has prevented the adoption by this community of normal software trends in bioinformatics, like automatic software deployment or the use of workflow managers. We have presented here the exercise of treating biomolecular simulations as normal bioinformatics operations. To this end, we have decorated standard simulation operations with a series of concepts and procedures, like an initial adherence to FAIR principles, the usage and documentation of workflows and stable interfaces, and the availability of a variety of deployment options, that are becoming routine in bioinformatics. FAIR principles for software have not yet been defined in the way as they exist for data. The exercise has led to an approach to the selection of software features (registration, methods of installations and deployment, documentation, licenses) that can be considered as an initial approach to them. The main outcome of the exercise is a complete software library (the BioBBs) that can be installed, deployed, and used as traditional bioinformatics applications, but provides a set of operations related to biomolecular simulations. BioBBs have been incorporated to the bioinformatics ecosystem: (1) The necessary new terms have been added to EDAM ontology, and tools included in the bio.tools registry. Bio.tools would provide a permanent identifier for them and the required metadata to assure their findability; (2) Interoperability has been addressed by the design of BioBBs architecture, but also through the use of recommended standards for specification (OpenAPI, CWL); and (3) Accessibility and usability have been addressed by using the set of well-known utilities, like Pipy, BioConda, BioContainers, or Galaxy, allowing the deployment and test of the library in a variety of alternative environments, from personal workstations to HPC.

BioBBs align with a variety of software that focus in similar functionality, however it opens the integration of biomolecular simulation operations into a more general bioinformatics landscape using similar, and compatible, software management procedures.

## Methods

### Atomistic simulations

#### Lysozyme test

Simulation of two sequence variants (Val2Tyr, and Val2Ala) of chicken Lysozyme (PDB code 1AKI)^46^ were prepared as follows. Protein structure was obtained from the Protein Data Bank^49^. Amino acid side chains were modified as appropriate using the biobb_model package. Hydrogen atoms were added to the structure using standard ionization at pH 7.0. Protein was placed in a Cubic box of explicit water solvent (SPC/E water model)^50^ with the appropriate size to allow 1 nm from the outermost protein atom. Periodic Boundary Conditions were applied. Cl^−^ and Na^+^ ions were added to reach an ion concentration of 0.05 M and neutralize the system. Simulations were run using GROMACS 2018, and the Amber99sb-ILDN forcefield^51^. Temperature was maintained at 300 K and pressure to 1 atm. Setup was completed by 5,000 steps of steepest-descent energy minimization, followed by a 10 ps-long NVT equilibration, and a 10 ps-long NPT equilibration runs with a restriction of 1,000 kJ/mol.nm^2^ put on heavy atoms. Production phase for the test consisted in 5 ns of unbiased NPT simulation at 2 fs time step. The LINCS algorithm^52^ was used to keep covalent bonds at their equilibrium distances. Simulation setup and equilibration were done using components of the biobb_md package.

#### Pyruvate kinase test

200 sequence variants for Human erythrocyte Pyruvate kinase (PDB code 2VGB)^47^ were obtained from UniprotKB^53^ (biobb_io package). Protein structure was obtained from the Protein Data Bank^49^. All non-protein components of the structure were removed, and protein variants were prepared by modification of the appropriate amino acid side chains using biobb_model package. Hydrogen atom were added considering standard ionization states at pH 7.0. Simulation was done in a truncated octahedron box placed at a distance of 1.5 nm from the outermost atom of the protein, using TIP3P water molecules^54^, and using Periodic Boundary Conditions. Ions Cl^−^ and Na^+^ were added to reach an ion concentration of 0.05 M and neutralize the system. The Particle mesh Ewald method^55^ was used to calculate electrostatic and Van der Waals interactions, with 0.12 nm of FF grid spacing and a cut-off distance of 1 nm for both Coulomb and Lennard-Jones interactions. The LINCS algorithm^52^ was used to keep covalent bonds at their equilibrium distances. Simulations were run using GROMACS 2018, and the Amber99sb-ILDN forcefield^51^. Temperature was maintained constant at 300 K (except in gradual heating), in two separate baths for the protein and non-protein groups, with the V-rescale thermostat^56^ and a coupling constant of 0.1 ps. Pressure was isotropically maintained at 1 bar in NPT ensembles through Parrinello-Rahman coupling^57^ with a constant of 1 ps, and applying a scaling of the center of mass of the reference coordinates with the scaling matrix. Given the size and complexity of the system, the Pyruvate kinase equilibration was performed with a more extended procedure: Setup was completed with two 5,000 steps energy minimizations, the first with a restrained potential of 500 kJ.mol^−1^.nm^−2^ on all heavy atoms except those in the side chain of the mutated residue, and the second with all heavy atoms restrained. Systems were then equilibrated with the following steps: (1) 100 ps of gradual heating from 0 to 300 K with 1,000 kJ.mol^−1^.nm^−2^ of restrained potential in heavy atoms except for mutated side chains, (2) four 20 ps steps of equilibration with descending restrain force constants in the same atoms (from 1,000 to 300 kJ.mol^−1^.nm^−2^), (3) two 10 ps steps of NPT equilibration with restraints in all backbone atoms (200 and 100 kJ.mol^−1^.nm^−2^ respectively) and (4) a 100 ps NPT equilibration without restraints. After equilibration, we ran 5 ns of unbiased NPT simulation. Simulation setup and equilibration were done using components of the biobb_md package.

### Computational systems used

Systems used on Lysozyme test were: *Workstation:* ThinkStation E30 (LENOVO). Operating system: Linux Ubuntu 18.04. 8 CPU Intel(R) Xeon(R) CPU E31230 @ 3.20 GHz (1 socket, 4 cores/socket, 2 threads/core). 16 GB of RAM. *Virtual Machine:* 12 CPU QEMU Virtual CPU version 2.5+. 24 GB of RAM. *Galaxy:* 2 CPU QEMU Virtual CPU version 2.5+. Pyruvate kinase test was performed on BSC’s MareNostrum supercomputer using from 2 to 800 nodes of 2x Intel Xeon Platinum 8160 24C at 2.1 GHz, 12 × 8 GB of RAM. Largest test used 4 nodes per simulation with a total of 38,400 cores.

## Supplementary information

Supplementary information.

## Data Availability

The test data of each building block is available in the correspondent Github repository, see Online-only Table 1. The full data collection on the testing phase for BioBBs is available at ref.^58^.

## Online-only Table

**Online-only Table 1:** URLs to BioBB’s distribution sites.

Software	Site type	Link
biobb (general)	Documentation	https://biobb.readthedocs.io
	Source code	https://github.com/bioexcel/biobb
biobb_io	Documentation	https://readthedocs.org/projects/biobb-io/
	Source code	https://github.com/bioexcel/biobb_io
	Package (PyPi)	https://pypi.org/project/biobb-io/
	Package (Bioconda)	https://anaconda.org/bioconda/biobb_io
	Container (Docker)	https://quay.io/repository/biocontainers/biobb_io
	Container (Singularity)	https://cloud.sylabs.io/library/andriopau/default/biobb
biobb_model	Documentation	https://biobb-model.readthedocs.io
	Source code	https://github.com/bioexcel/biobb_model
	Package (PyPi)	https://pypi.org/project/biobb-model/
	Package (Bioconda)	https://anaconda.org/bioconda/biobb_model
	Container (Docker)	https://quay.io/repository/biocontainers/biobb_model
	Container (Singularity)	https://cloud.sylabs.io/library/andriopau/default/biobb
biobb_md	Documentation	https://biobb-md.readthedocs.io
	Source code	https://github.com/bioexcel/biobb_md
	Package (PyPi)	https://pypi.org/project/biobb-md/
	Package (Bioconda)	https://anaconda.org/bioconda/biobb_md
	Container (Docker)	https://quay.io/repository/biocontainers/biobb_md
	Container (Singularity)	https://cloud.sylabs.io/library/andriopau/default/biobb
biobb_analysis	Documentation	https://biobb-analysis.readthedocs.io
	Source code	https://github.com/bioexcel/biobb_analysis
	Package (PyPi)	https://pypi.org/project/biobb-analysis/
	Package (Bioconda)	https://anaconda.org/bioconda/biobb_analysis
	Container (Docker)	https://cloud.docker.com/u/mmbirb/repository/docker/mmbirb/biobb_analysis
	Container (Singularity)	https://cloud.sylabs.io/library/andriopau/default/biobb
biobb_common	Documentation	https://biobb-common.readthedocs.io
	Source code	https://github.com/bioexcel/biobb_common
	Package (PyPi)	https://pypi.org/project/biobb-common/
	Package (Bioconda)	https://anaconda.org/bioconda/biobb_common
	Container (Docker)	https://quay.io/repository/biocontainers/biobb_common
biobb_template	Documentation	https://biobb-template.readthedocs.io
	Source code	https://github.com/bioexcel/biobb_template
biobb_adapters	Source code	https://github.com/bioexcel/biobb_adapters
biobb_wf_mutations	Source code	https://github.com/bioexcel/biobb_wf_mutations
	Package (PyPi)	https://pypi.org/project/biobb-wf-mutations/
	Jupyter Notebook	https://mybinder.org/v2/gh/bioexcel/biobb_workflows/master
	Galaxy	https://dev.usegalaxy.es/workflows/list_published
	Virtual Machine	http://mmb.irbbarcelona.org/BioExcel/biobb.ova

## Code Availability

BioBB’s source code is available at GitHub. URLs for the code and documentation repositories and the alternative installation and execution options are summarized in Online-only Table 1.
