# Supplementary material for: BioExcel Building Blocks, a software library for interoperable biomolecular simulation workflows
Source: Sci Data. 2019 Sep 10;6:169. doi: 10.1038/s41597-019-0177-4 (PMC6736963; doi:10.1038/s41597-019-0177-4)
Supplement: Supplementary file 1 — Supplementary information. [file 41597_2019_177_MOESM1_ESM.docx]

**BioExcel Building Blocks, a software library for interoperable biomolecular simulation workflows.**

Pau Andrio (1), Adam Hospital (2), Javier Conejero (1), Luis Jordà (1), Marc Del Pino (1), Laia Codo (1), Stian Soiland-Reyes (3), Carole Goble (3), Daniele Lezzi (1), Rosa M. Badia (1), Modesto Orozco (2,4), Josep LL. Gelpi (1,4).

1. Barcelona Supercomputing Center (BSC). Jordi Girona 29 08034 Barcelona, Spain.
2. Institute for Research in Biomedicine (IRB Barcelona), The Barcelona Institute of Science and Technology (BIST), Baldiri Reixac 10, Barcelona, 08028, Spain.
3. School of Computer Science, The University of Manchester, Manchester, United Kingdom.
4. Dept. Biochemistry and Molecular BIomedicine, University of Barcelona, Spain.

**Supplementary Figures and Tables:**

Figure S1: Workflow schema for the molecular dynamics simulation setup used as test case.
Workflow performs a complete setup and equilibration of a series of simulations of aprotein system with sequence variants, followed by a simple RMSd analysis.

Figure S2: Workflow schema as implemented in the Galaxy GUI.

Table S1: Additional terms added to EDAM ontology to cover Biomolecular simulations.

Table S2: Example of code-embedded information used to generate BioBBs documentation.
Python docstrings are used to include documentation specific for library classes and methods. Documentation is captured by Sphinx.

Table S3: Test execution cases for BioBB.
Data can be obtained from http://doi.org/10.5281/zenodo.2581362.


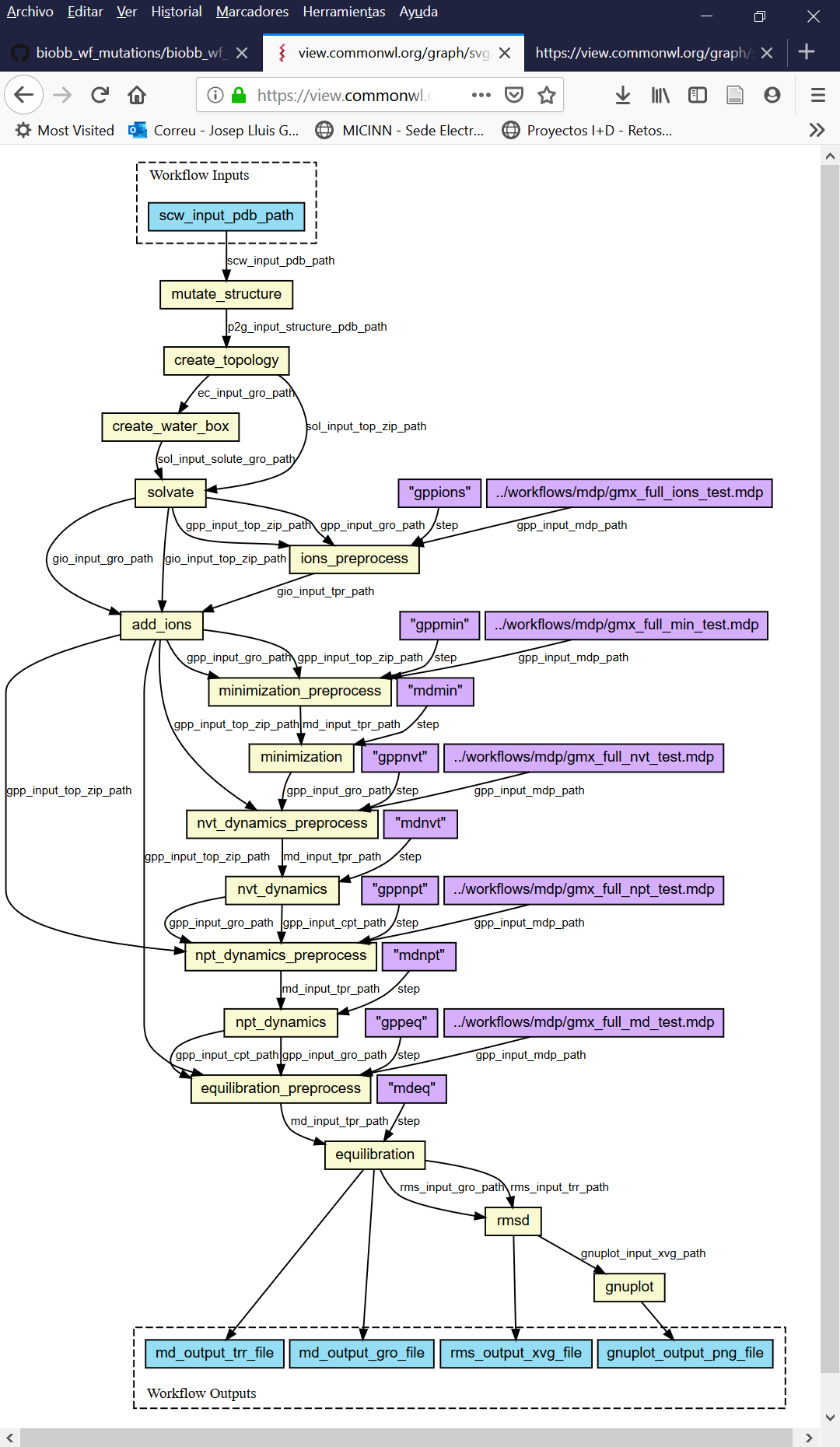


Figure S1: Workflow schema for the molecular dynamics simulation setup used as test case.
Workflow performs a complete setup and equilibration of a series of simulations of a protein system with sequence variants, followed by a simple RMSd analysis.


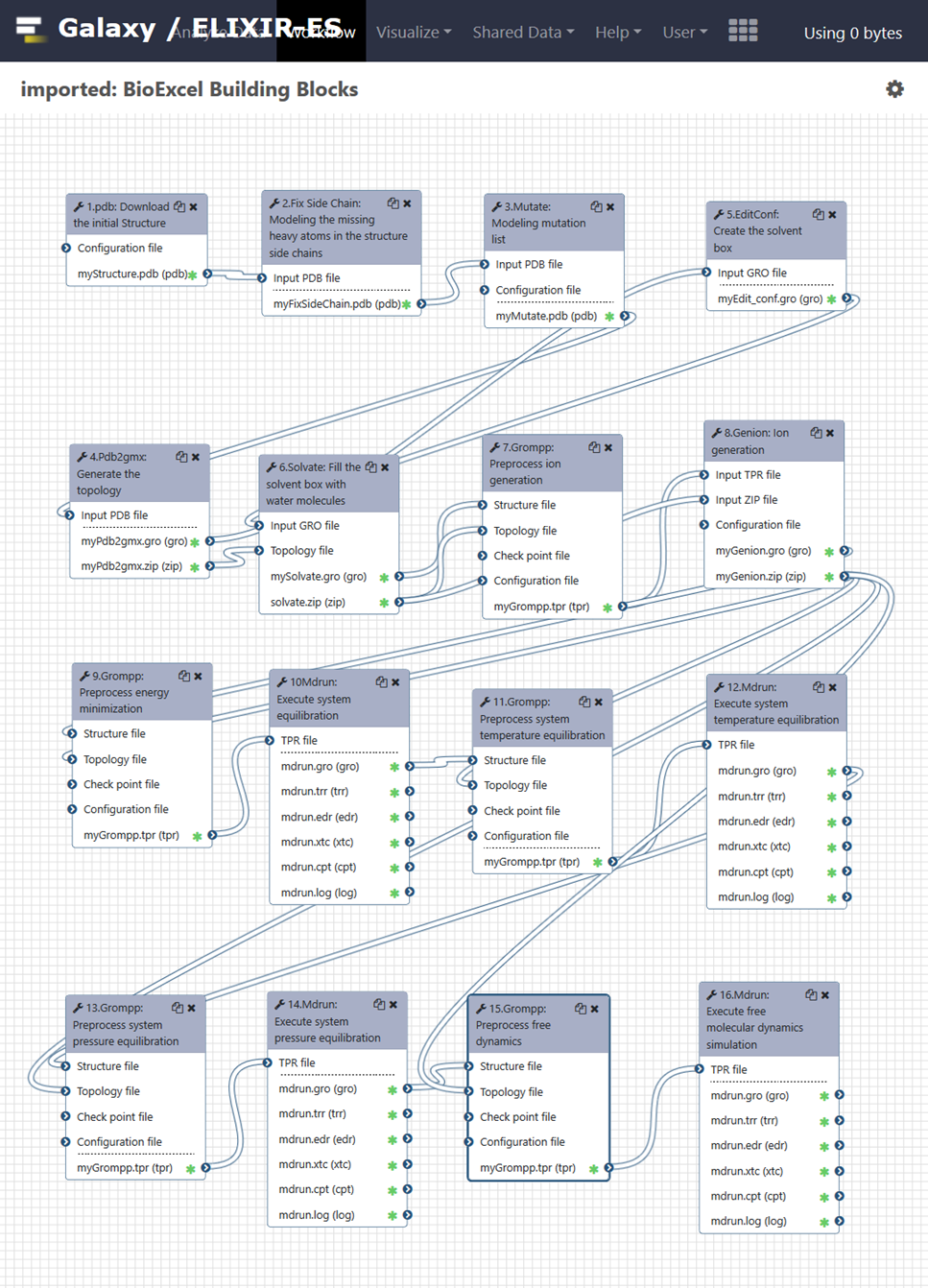


Figure S2: Workflow schema as implemented in the Galaxy GUI.

Table S1: Additional terms added to EDAM ontology to cover Biomolecular simulations.

| **Section** | **Class** | **Description** | **EDAM term** |
| --- | --- | --- | --- |
| TOPIC | Biomolecular Simulation | The study and simulation of molecular conformations using a computational model and computer simulations. | 3892 |
| OPERATION | Forcefield parameterization | Obtain force field parameters from a molecule, to be used in molecular simulations | 3893 |
|  | Simulation analysis | Analysis of molecular simulation trajectory data | 0244 |
|  | Trajectory visualization | 3D visualization of a molecular trajectory | 3890 |
|  | Essential Dynamics | Analysis of molecule dynamics using PCA (Principal Component Analysis) applied to the atomic positional fluctuations. | 3891 |
| DATA | Molecular Simulation data | Data coming from molecular simulations. Typically formed by two separated but indivisible pieces of information: topology data (static) and trajectory data (dynamic). | 3842 |
|  | Topology data | Static information of a structure molecular system that is needed for a molecular simulation: the list of atoms, their non-bonded parameters for Van der Waals and electrostatic interactions, and the complete connectivity in terms of bonds, angles and dihedrals. | 3872 |
|  | Trajectory data | Dynamic information of a structure molecular system coming from a molecular simulation: XYZ 3D coordinates (sometimes with their associated velocities) for every atom along time. | 3870 |
|  | Forcefield parameters | Force field parameters: charges, masses, radii, bond lengths, bond dihedrals, etc. define the structural molecular system, and are essential for the proper description and simulation of a molecular system. | 3871 |
| FORMAT | Trajectory format | File formats to store 3D-structure's trajectory information. | 3866 |
|  | XTC | Portable binary format for trajectories produced by GROMACS package. | 3875 |
|  | TNG | Trajectory Next Generation (TNG) is a format for storage of molecular simulation data. It is designed and implemented by the GROMACS development group, and it is called to be the substitute of the XTC format. | 3876 |
|  | BinPos | Scripps Research Institute’s BinPos format is a binary formatted file to store atom coordinates. | 3885 |

Table S2: Example of code-embedded information used to generate BioBBs documentation.
Python docstrings are used to include documentation specific for library classes and methods. Documentation is captured by Sphinx.

| class Grompp():  """Wrapper of the GROMACS grompp module.  The GROMACS preprocessor module needs to be feeded with the input system  and the dynamics parameters to create a portable binary run input file TPR.  The dynamics parameters are specified in the mdp section of the  configuration YAML file. The parameter names and defaults are the same as  the ones in the official MDP specification: http://manual.gromacs.org/current/online/mdp_opt.html    Args:  input_gro_path (str): Path to the input GROMACS structure GRO file.  input_top_zip_path (str): Path the input GROMACS topology TOP and ITP files in zip format.  output_tpr_path (str): Path to the output portable binary run file TPR.  input_cpt_path (str)[Optional]: Path to the input GROMACS checkpoint file CPT.  properties (dic):  \| - **input_mdp_path** (*str*) - (None) Path of the input MDP file.  \| - **mdp** (*dict*) - (defaults dict) MDP options specification. (If *input_mdp_path* is None)  \| - **type** (*str*) - ("minimization") Options for the mdp file. Values: nvt, npt, free, index.  \| - **output_mdp_path** (*str*) - ("grompp.mdp") Path of the output MDP file.  \| - **output_top_path** (*str*) - ("grompp.top") Path the output topology TOP file.  \| - **maxwarn** (*int*) - (10) Maximum number of allowed warnings.  \| - **gmx_path** (*str*) - ("gmx") Path to the GROMACS executable binary.  """ |
| --- |

Table S3: Test execution cases for BioBB.
Data can be obtained from http://doi.org/10.5281/zenodo.2581362.

| **Execution system** | **Numberof cores** | **Simulated system** | **System atom number** | **Simulation time** | **Number of simulated mutations** | **Execution type** | **Execution time** |
| --- | --- | --- | --- | --- | --- | --- | --- |
| Workstation | 8 | Lysozyme (1AKI) | 33,838 | 5 ns | 2 | Serial | 1,184 min |
| OpenNebula VM | 12 | Lysozyme (1AKI) | 33,838 | 5 ns | 2 | Serial | 559 min |
| Galaxy public server | 2 | Lysozyme (1AKI) | 33,838 | 5ns | 1 | Serial | ~1,500 min |
| MareNostrum 4 | 384 | Pyruvate Kinase (2VGB) | 395,415 | 5 ns | 2 | Parallel PyCOMPSs | 244 min |
| MareNostrum 4 | 38400 | Pyruvate Kinase (2VGB) | 395,415 | 5 ns | 200 | Parallel PyCOMPSs | 317 min |
